# Long-term physical activity in middle-aged patients with meniscal symptoms: a 10-year follow-up of a randomised controlled trial comparing exercise therapy alone to knee arthroscopic surgery plus exercise therapy

**DOI:** 10.1186/s12891-025-09151-4

**Published:** 2025-09-23

**Authors:** Sofi Sonesson, Roman Peter Kuster, Håkan Gauffin, Joanna Kvist

**Affiliations:** 1 Unit of Physiotherapy, Department of Medical and Health Sciences, Linköping University, Campus US, entrance 78, level 15, Linköping, S-581 83 Sweden; 2 Therapy Science Lab, Lake Lucerne Institute, Vitznau, Switzerland; 3 Division of Physiotherapy, Department of Neurobiology, Care Sciences and Society, Karolinska Institutet, Huddinge, Sweden; 4 Vascular Neurology and Neurorehabilitation, Department of Neurology, University of Zurich and University Hospital Zurich, Zurich, Switzerland; 5 Department of Orthopedics and Department of Clinical and Experimental Medicine, Linköping University, Linköping, Sweden

**Keywords:** Accelerometry, Physical activity, Knee injury, Knee pain, Knee surgery, Long-term outcome

## Abstract

**Background:**

Physical activity levels in individuals with a degenerative meniscus lesion and the impact of different treatment approaches remain unclear. The purpose was to compare sensor-based measurements of physical activity and self-reported physical activity at 10-year follow-up of a randomised controlled trial between individuals with meniscal symptoms allocated to (1) exercise therapy or (2) knee arthroscopy combined with exercise therapy, and to analyse correlations between physical activity and a) patient-reported outcomes, b) functional performance.

**Methods:**

Patients aged 45 to 64 years with meniscal symptoms were randomised to exercise therapy (non-surgery group), or knee arthroscopy combined with exercise therapy (surgery group). The physical activity of 62 patients (30 in non-surgery group and 32 in surgery group) was assessed using an activPAL accelerometer for 7 days and the Physical Activity Scale at the 10-year follow-up. Patients answered the Knee injury and Osteoarthritis Outcome Score (KOOS) and EuroQol quality of Life assessment. Functional performance was assessed with the 30-second chair stand test. Intention-to-treat was the primary analytic approach.

**Results:**

Accelerometer-assessed physical activity did not differ between treatment groups. High to moderate physical activity was more frequently reported in the non-surgery group compared to the surgery group (66% vs. 34%, *p* = .015) (Intention-to-treat). As-treated analysis showed no group differences. Accelerometer measured steps per day and self-reported physical activity correlated positively with the 30-second chair stand test (*r* = .336, *p* = .008; *r* = .423, *p* < .001). Self-reported physical activity correlated positively with KOOS_PAIN_, KOOS_ADL_ and KOOS_SPORT_ *r* = .259 − .375, *p* ≤ .044).

**Conclusions:**

Sensor-based physical activity levels did not differ between the treatment groups. However, higher self-reported physical activity was associated with less pain and better function in daily activities as well as in sports and recreational pursuits. Furthermore, participants who walked more steps per day and reported higher levels of physical activity demonstrated better functional performance. These findings highlight the importance of promoting physical activity in this population to enhance knee health and overall quality of life.

**Trial registration:**

Clinical Trials NCT01288768, retrospectively registered 2011-02-01.

## Introduction

Degenerative meniscus lesions are prevalent among middle-aged individuals [1]. Randomised controlled trials have evaluated the efficacy of arthroscopic partial meniscectomy for these patients and a systematic review concluded that arthroscopic knee surgery offers minimal to no clinically significant benefits in terms of pain or functional improvement [2]. Yet, some studies have reported a modest positive effect on pain relief lasting up to one [3, 4] or two years [5]. In our prospective randomised controlled trial, we observed a one-year benefit from combining knee arthroscopic surgery with exercise therapy compared to exercise therapy alone in middle-aged patients with meniscus symptoms [6]. At the 10-year follow-up, no differences were found between the groups regarding knee pain, symptoms, radiographic findings or clinical status [7]. The finding that the treatments did not differ in terms of osteoarthritis (OA) progression at 10 years was recently confirmed in another trial [8]. 

The physical activity of individuals with a degenerative meniscus lesion might be reduced due to knee pain, knee symptoms and functional limitations. However, long-term physical activity levels following degenerative meniscus lesions and their association with pain and function remain unknown. The impact of different treatment approaches for meniscus injuries on long-term physical activity levels is also unclear. Physical activity is crucial for maintaining health and preventing chronic diseases such as cardiovascular disease, diabetes and obesity [9, 10]. Physical inactivity in people with knee OA leads to substantial loss of quality-adjusted life-years [11]. In individuals with knee OA, higher physical activity levels are associated with less knee pain, although a meta-analysis found conflicting associations between physical activity and pain [12]. Understanding the factors associated with high physical activity could improve targeted interventions to support healthy physical activity behaviour. Assessing physical activity using accelerometry consistently provides detailed information regarding intensity and duration [13], mitigates biases such as recall and social desirability, and accurately detects various physical activities with high sensitivity and specificity [14, 15]. 

To address current knowledge gaps, this study aimed to compare sensor-based measurements of physical activity and self-reported physical activity at the 10-year follow-up of a randomised controlled trial between individuals with meniscal symptoms allocated to (1) exercise therapy or to (2) knee arthroscopy combined with exercise therapy. A secondary aim was to analyse the correlations between physical activity and a) patient-reported outcomes, and b) functional performance. We hypothesised that there would be no difference between the two treatment groups in physical activity levels, and that physical activity levels would be positively correlated with patient-reported outcomes and functional performance.

## Methods

### Study design and participants

**Fig. 1:**
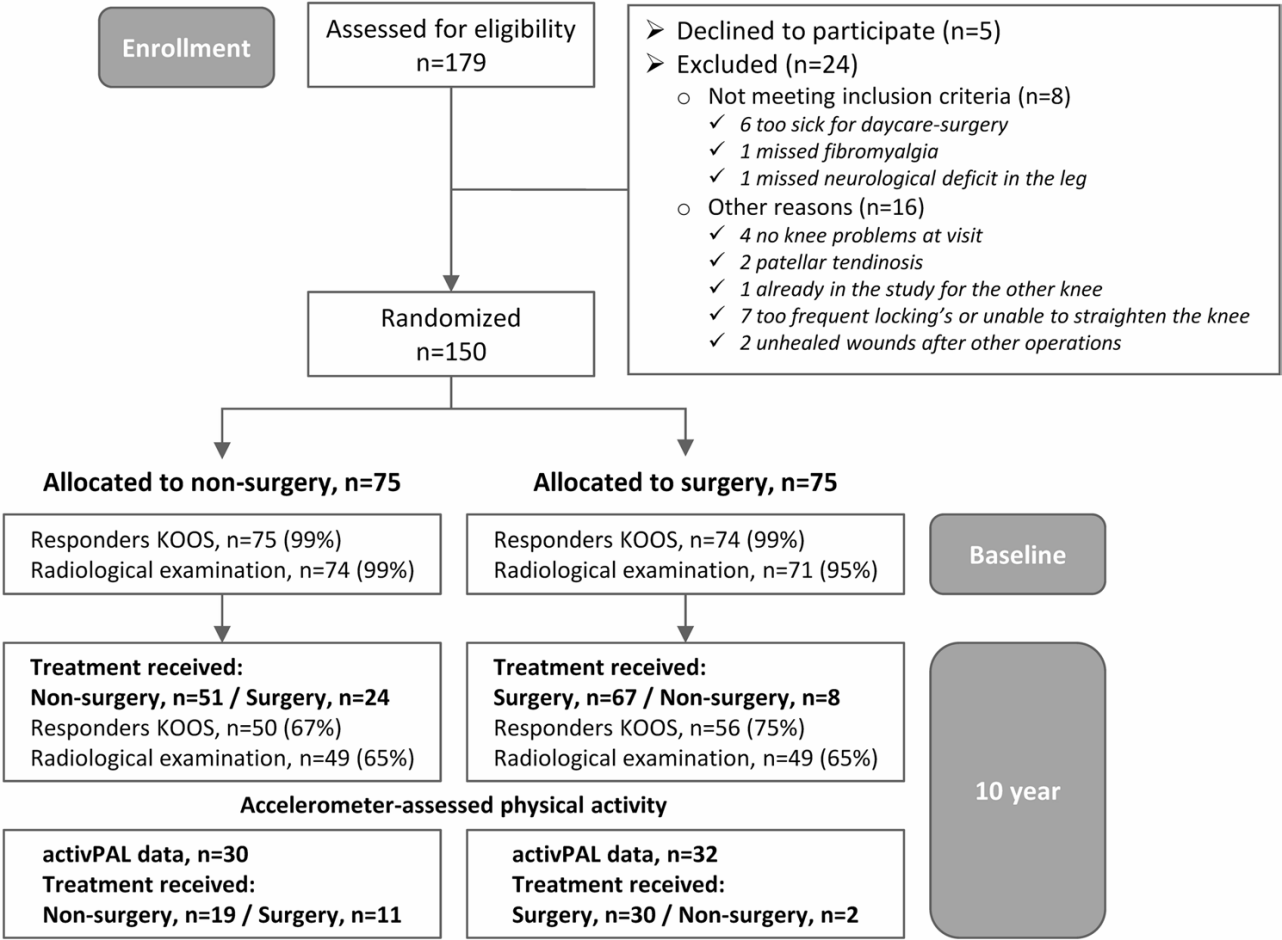
Flowchart of participants. Footnote: For the intention-to-treat analyses, 30 participants were included in the non-surgery group and 32 participants were included in the surgery group. For the as-treated analyses, 21 participants were included in the non-surgery group and 41 participants were included in the surgery group

This 10-year follow-up builds on a randomized controlled trial involving individuals with meniscal symptoms. Details concerning study design, sample size, and participant characteristics have been published previously [6, 7, 16, 17]. In brief, participants were recruited from the orthopaedic department at the Linköping University Hospital between 2010 and 2012. Inclusion criteria included age 45–64 years, knee symptoms for more than 3 months, Ahlbäck score 0 on standing X-ray [18], and at least three months’ of exercise therapy treatment. The clinical routine included exercise therapy for 3 months before referral to the orthopaedic department. Exclusion criteria included locked knee or joint locking for longer than 2 s more than once a week, rheumatic or neurological diseases, fibromyalgia, hip or knee replacements, or contraindications for day surgery (Fig. 1). The study was approved by the Ethics Committee at Linköping University (Dnr: 2010/6–31) and by the Swedish Ethical Review Authority (Dnr: 2020–04157). The study design and reporting follow the CONSORT (Consolidated Standards of Reporting Trials) guidelines for randomized trials.

### Randomisation and interventions

Patients were randomly assigned to:

1. Non-surgery: Physiotherapy assessment and exercise instructions within 2 weeks.
2. Surgery: Same physiotherapy assessment and exercise instructions plus knee arthroscopy within 4 weeks; significant meniscal injuries were resected.

Baseline data were collected pre-randomisation. The 3-month exercise program focused on muscle function and postural control. Surgeries were performed by experienced arthroscopists, and the decision to perform a meniscal resection was made by the surgeons based on their professional judgment.

### 10-year follow-up

Patients completed questionnaires, radiological exams, clinical assessments and wore an activPAL accelerometer for 7 days. Details of the 10-year follow-up methodology and results on the prevalence of radiographic OA, symptomatic OA, patient-reported outcomes and clinical status have been reported previously [7]. To be eligible for the present analyses, participants were required to have worn an accelerometer for at least 3 days at the 10-year follow-up and provided data on symptomatic OA. One participant was excluded due to missing data on symptomatic OA.

## Outcome measures

### Physical activity measures

#### Accelerometry

Sensor-based physical activity was assessed at the 10-year follow up. An activPAL accelerometer (PAL Technologies Ltd., Glasgow, SCO) was placed on the front thigh of the patient’s index leg following manufacturer recommendation. Patients were instructed to wear the activPAL device continuously for 7 days, 24 h a day.

Valid accelerometer data was provided by 30 participants in the non-surgery group and 32 participants in the surgery group, whereof 93% and 89% respectively provided data for ≥ 6 days. The processing of the activPAL data is described in Appendix 1 and has previously been explained in more detail [19]. 

#### Self-reported physical activity

The Six-level Saltin-Grimby Physical Activity Level Scale was used to evaluate self-reported physical activity with scores ranging from 1 to 6, where higher scores indicate greater physical activity levels [20, 21]. 

### Patient-reported outcome measures

Patients completed patient-reported outcome measures (PROMS) at baseline and at the 10-year follow-up. The Tegner Activity Scale assessed activity level, ranging from 0 (sick leave or disability due to knee problems) to 10 (competitive sports at the national elite level) [22]. The Knee Injury and Osteoarthritis Outcome Score (KOOS) evaluated five domains: pain, symptoms, activities of daily living (ADL), sports and recreation function, and knee-related quality of life (QoL). Each subscale is scored from 0 to 100, with 100 representing no problems and 0 indicating extreme problems [23]. The EuroQol Quality of Life Questionnaire (EQ-5D-3 L index) was used to measure health status across five dimensions: mobility, self-care, usual activities, pain/discomfort and anxiety/depression. A score of 1 indicates full health [24]. 

### Functional performance

Functional performance was evaluated at the 10-year follow-up. One of two orthopaedic surgeons assessed the 30-second chair stand test. In this test, patients were instructed to perform as many stands as possible within 30 s, ensuring they fully sat down between each stand. The test was conducted on two legs and results were recorded as the maximum number of stands.

### Radiographic assessment

Weight-bearing radiographs were taken at baseline and at the 10-year follow-up. A single radiologist, who was blinded to the allocation and treatments, evaluated all x-rays according to the Kellgren & Lawrence (KL) score [25]. The grades were as follows:

- Grade 1: possible osteophytes only
- Grade 2: definite osteophytes and possible joint space narrowing
- Grade 3: moderate osteophytes and/or definite narrowing
- Grade 4: large osteophytes, severe joint space narrowing, and/or bony sclerosis

Radiographic OA was defined as Grade 2 or higher [26]. Progression of radiographic OA was defined having at least 1-step deterioration compared to the baseline measurement and having a KL Grade of 2 or higher. Symptomatic OA was defined as the presence of radiographic OA in the tibiofemoral joint together with the presence of knee pain and/or symptoms defined as a ≥ 1-step reduction from the maximum score (no knee symptoms) to ≥ 50% of items within the KOOS Pain and/or Symptoms subscales [27]. 

### Patient and public involvement

Patients and the public were not involved in the design or conduct of this study.

### Statistical analysis

Descriptive statistics of continuous data are presented as mean ± standard deviations (SD), and data on the Tegner activity scale are presented as median (interquartile range (IQR)). Nominal data are displayed as the number of patients and percentages. An intention-to-treat approach was used as the primary analyses throughout, and as-treated analyses were also performed. For the as-treated analyses, patients that underwent knee arthroscopic surgery in the index knee between their allocation and the 1-year follow-up were included in the surgery group. Patients that did not undergo knee arthroscopic surgery in the index knee during the first year after inclusion were included in the non-surgery group.

Analysis of covariance (ANCOVA) was used to assess differences in the physical activity parameters between treatment groups, adjusting for symptomatic OA as a potential confounding factor.

The percentage of individuals not reaching the 150 min of MVPA per week and 300 min of MVPA per week according to World Health Organization’s physical activity recommendations was calculated using total MVPA [28]. 

Pearson’s product-moment correlation coefficient (r) was used to analyse linear correlations between MVPA, steps per day, Physical Activity Level Scale, and patient reported outcome measures (KOOS and EQ-5D-3 L index) and 30-second chair stand tests. Strength of correlation coefficients were interpreted as follows: negligible ≤ 0.30, weak 0.31–0.50, moderate 0.51–0.70, and strong > 0.71 [29]. 

A drop out analysis was conducted to control for differences between participants in the current activPAL cohort (*n* = 62) and those from the original cohort not included in the present study (*n* = 88). Baseline group differences in age and KOOS were analysed using independent samples t-test, differences in physical activity, sex, radiographic OA, and symptomatic OA were analysed using chi-square test, and difference in EQ-5D-3 L index was analysed with Mann-Whitney test.

All statistical analyses were performed with IBM SPSS Statistics for Windows (Version 29.0. Armonk, NY: IBM Corp). The significance level was set at 5% for all analyses.

## Results

### Patient characteristics

At the 10-year follow-up, 62 patients—30 in the non-surgery group and 32 in the surgery group—provided valid accelerometer data and data on self-reported outcomes. The baseline characteristics of this cohort, including the Tegner activity level and KOOS score, were similar between the groups, although radiographic OA was more prevalent in the non-surgery group (57% vs. 27%, *p* =.018) (Table 1). At the 10-year follow-up, there were no significant differences between treatment groups in patient characteristics, number of knee surgeries, radiographic OA or symptomatic OA (Table 2).

**Table 1:** Patient characteristics, self-reported function and self-reported physical activity at baseline by intention-to-treat at 10 years (*n* = 62)

	Intention-to-treat			
	Total cohort	Non-surgery	Surgery	*p*-value
	*n* = 62	*n* = 30	*n* = 32	
Tegner activity level, median (IQR)	3 (2–4)^5^	3 (1.5–4)^2^	3 (2–4)^3^	.380
Moderate to high physical activity (PAS 4–6) during last month, *n* (%)	30 (48%)	15 (50%)	15 (47%)	.806
KOOS symptoms, mean ± SD	59.9 ± 17.3^1^	61.8 ± 19.4^1^	58.1 ± 15.4	.401
KOOS pain, mean ± SD	59.0 ± 14.8	59.8 ± 17.5	58.2 ± 11.9	.675
KOOS ADL, mean ± SD	69.6 ± 16.2^1^	70.8 ± 17.9^1^	68.5 ± 14.7	.575
KOOS sport and recreational, mean ± SD	31.1 ± 20.7^1^	32.2 ± 23.1	30.2 ± 18.6^1^	.709
KOOS QoL, mean ± SD	35.0 ± 14.1	35.1 ± 15.7	35.0 ± 12.7	.978
Radiographic osteoarthritis, *n* (%)	25 (42%)^2^	17 (57%)^1^	8 (27%)^1^	.018
KL grade 0	20 (33%)	7 (23%)	13 (43%)	
KL grade 1	15 (25%)	6 (20%)	9 (30%)	
KL grade 2	25 (42%)	17 (57%)	8 (27%)	

Superscript numbers indicate missing values

*IQR* inter quartile range, *KL* Kellgren & Lawrence score, *KOOS* knee injury and osteoarthritis outcome score, *SD* standard deviation

**Table 2:** Patient characteristics, self-reported function, self-reported physical activity and functional performance at 10 years by intention-to-treat at 10 years (*n* = 62)

	Intention-to-treat			
	Total cohort	Non-surgery	Surgery	*p*-value
	*n* = 62	*n* = 30	*n* = 32	
Age, years, mean ± SD	65.8 ± 5.5	65.9 ± 5.8	65.8 ± 5.3	.986
Males, *n* (%)	30 (48%)	16 (53%)	14 (44%)	.450
BMI (kg/m^2^), mean ± SD	25.2 ± 3.4	25.6 ± 3.3	24.7 ± 3.4	.305
BMI classes, *n* (%)				.819^F^
18.5–24.9 Normal weight	33 (53%)	15 (50%)	18 (56%)	
25.0–29.9 Overweight	22 (35%)	11 (37%)	11 (34%)	
30.0–34.9 Obesity class I	7 (11%)	4 (13%)	3 (9%)	
KOOS symptoms, mean ± SD	73.0 ± 22.2^1^	77.1 ± 19.8^1^	69.2 ± 23.8	.167
KOOS pain, mean ± SD	76.8 ± 19.9^1^	81.0 ± 17.6^1^	73.0 ± 21.3	.114
KOOS ADL, mean ± SD	81.7 ± 19.1^1^	85.6 ± 16.3^1^	78.2 ± 21.0	.133
KOOS sport and recreational, mean ± SD	48.1 ± 29.5^2^	52.9 ± 28.0^1^	43.6 ± 30.6^1^	.225
KOOS QoL, mean ± SD	60.7 ± 25.7^1^	64.0 ± 25.6^1^	57.6 ± 25.8	.337
Radiographic osteoarthritis, *n* (%)	42 (68%)	21 (70%)	21 (66%)	.713
Symptomatic osteoarthritis, *n* (%)	31 (50%)	13 (43%)	18 (56%)	.309
Total number of surgeries to index knee, *n* (%)				n/a
0	20 (32%)	19 (63%)	1 (3%)	
1	37 (60%)	9 (30%)	28 (88%)	
2	5 (8%)	2 (7%)	3 (9%)	
Total number of surgeries to contralateral knee, *n* (%)				.197^F^
0	53 (85%)	28 (93%)	25 (78%)	
1	8 (13%)	2 (7%)	6 (19%)	
2	1 (2%)	0 (0%)	1 (3%)	
Comorbidity, number of other conditions in health declaration, *n* (%)				.894^F^
0 conditions	37 (60%)	18 (60%)	19 (59%)	
1 condition	24 (39%)	11 (37%)	13 (41%)	
2–4 conditions	1 (2%)	1 (3%)	0 (0%)	
Current or former smoker or snuff user, *n* (%)	24 (39%)^1^	9 (30%)	15 (48%)^1^	.142
Education, university level, *n* (%)	27 (44%)^1^	13 (43%)	14 (45%)^1^	.886
Employment, *n* (%)				.076
Employed/Job seeker	30 (48%)	18 (60%)	12 (38%)	
Retired/Other	32 (52%)	12 (40%)	20 (63%)	
Employment, load, *n* (%)				.606
Light work	23 (37%)	13 (43%)	10 (31%)	
Mobile work	20 (32%)	9 (30%)	11 (34%)	
Heavy work	19 (31%)	8 (27%)	11 (34%)	
Wish you had performed some other type of exercise activity/sport, *n* (%)	32 (53%)^2^	18 (62%)^1^	14 (45%)^1^	.190
Acceptable knee status, *n* (%)	46 (78%)^3^	24 (86%)^2^	22 (71%)^1^	.172
EQ-5D-3 L index, mean ± SD	0.77 ± 0.18^2^	0.81 ± 0.12^2^	0.75 ± 0.21	.205
Moderate to high physical activity (PALS 4–6) during last month, *n* (%)	30 (49%)^1^	19 (66%)^1^	11 (34%)	.015
30-second chair stand test, mean ± SD	14.1 ± 5.4^1^	15.6 ± 4.8	12.7 ± 5.7^1^	.041

Superscript numbers indicate missing values

*F* Fishers exact test, *KOOS* knee injury and osteoarthritis outcome score, *PALS* physical activity level scale, *SD* standard deviation

### Accelerometer assessed physical activity

**Fig. 2:**
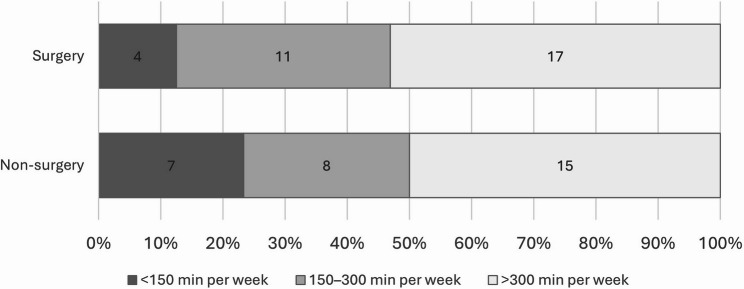
Accelerometer assessed moderate to vigorous physical activity average minutes per week cut-offs by intention-to-treat at 10 years

At the 10-year follow-up, patients in the non-surgery group spent an average of 42.7 min per day (95% CI 34.1–54.4) engaged in MVPA, while those in the surgery group spent an average of 53.1 min per day (95% CI 41.8–64.4) (*p* =.208). There were no significant differences in any accelerometer data variables between the treatment groups (Table 3). The threshold of ≥ 150 min per week of MVPA was achieved by 77% of patients in the non-surgery group and 88% of patients in the surgery group (Fig. 2).

**Table 3:** Accelerometer data at 10 years by intention-to-treat (*n* = 62)

activPAL	Intention-to-treat			
	Total cohort	Non-surgery	Surgery	*p*-value
	*n* = 62	*n* = 30	*n* = 32	
	Mean (95% CI)	Mean (95% CI)	Mean (95% CI)	
MVPA > 1 min				
average min per day	48.1 (40.0–56.1)	42.7 (31.1–54.4)	53.1 (41.8–64.4)	.208
% of day	3.3% (2.8–3.9)	3.0% (2.2–3.8)	3.7% (2.9–4.5)	
MVPA > 10 min				
average min per day	29.9 (22.4–37.5)	26.5 (15.6–37.4)	33.0 (22.5–43.6)	.397
% of day	2.1% (1.6–2.6)	1.8% (1.1–2.6)	2.3% (1.6–3.0)	
MVPA 1–10 min				
average min per day	18.2 (15.7–20.7)	16.2 (12.6–19.7)	20.1 (16.6–23.5)	.122
% of day	1.3% (1.1–1.4)	1.1% (0.9–1.4)	1.4% (1.2–1.6)	
LIPA				
average min per day	84.3 (76.9–91.8)	87.9 (77.2–98.5)	81.0 (70.7–91.4)	.364
% of day	5.9% (5.3–6.4)	6.1% (5.4–6.8)	5.6% (4.9–6.3)	
Standing				
average min per day	316.8 (290.6–343.0)	309.2 (272.2–346.1)	323.9 (288.2–359.7)	.568
% of day	22.0% (20.2–23.8)	21.5% (18.9–24.0)	22.5% (20.0–25.0)	
Sitting				
average min per day	534.9 (508.2–561.6)	542.2 (503.7–580.7)	528.1 (490.9–565.4)	.603
% of day	37.1% (35.3–39.0)	37.7% (35.0–40.3)	36.7% (34.1–39.3)	
Lying				
average min per day	455.9 (432.1–479.6)	458.1 (423.2–493.0)	453.8 (420.0–487.5)	.860
% of day	31.7% (30.0–33.3)	31.8% (29.4–34.2)	31.5% (29.2–33.9)	
Knee load				
average min per day	449.2 (419.7–478.8)	439.7 (397.3–482.2)	458.1 (417.0–499.2)	.539
% of day	31.2% (29.1–33.2)	30.5% (27.6–33.5)	31.8% (29.0–34.7)	
Steps per day	5,195 (4,739–5,651)	5,075 (4,413–5,738)	5,307 (4,666–5,949)	.618
Sitting episodes per day	48.1 (45.2–51.1)	48.4 (44.2–52.6)	47.9 (43.9–51.9)	.865

*CI* confidence interval, *LIPA* light intensity physical activity, *MVPA* moderate to vigorous physical activity

### Self-reported physical activity

At baseline, 50% of the patients in the non-surgery group and 47% of patients in the surgery group reported engaging in high to moderate physical activity (Physical Activity Level Scale 4–6; moderate exercise 1–2 h a week to strenuous exercise regularly several times a week) (*p* =.806).

At the 10-year follow-up, 66% of the patients in the non-surgery group reported engaging in high to moderate physical activity compared to 34% of patients in the surgery group (*p* =.015).

### Correlation between physical activity patterns, patient reported outcomes and functional performance

At the 10-year follow-up, the number of steps per day and self-reported physical activity correlated positively with the 30-second chair stand test (*r* =.338, *p* =.008; *r* =.423, *p* <.001). Additionally, self-reported physical activity showed positive correlations with the KOOS_PAIN_, KOOS_ADL_ and KOOS_SPORT and RECREATION_ (*r* =.259 to 0.375, *p* ≤.044). However, time spent engaged in MVPA did not correlate with PROMS such as the Physical Activity Level Scale, KOOS, or EQ-5D-3 L index or with the 30-second chair stand test (Table 4).

**Table 4:** Pearson correlation coefficients between sensor based and self-reported physical activity, and patient reported outcome measures and functional performance at 10 years (*n* = 62)

		MVPA > 1 min, average min per day	Steps per day	PAS 4–6
Steps per day	Pearson correlation	.857	–	
	*p*-value	< .001		
PALS 4–6	Pearson correlation	.097^1^	.238^1^	–
	*p*-value	.459	.065	
KOOS symptoms	Pearson correlation	−.023^1^	−.005^1^	.246^1^
	*p*-value	.859	.971	.056
KOOS pain	Pearson correlation	.045^1^	.078^1^	.259^1^
	*p*-value	.732	.549	.044
KOOS ADL	Pearson correlation	.032^1^	.114^1^	.295^1^
	*p*-value	.807	.380	.021
KOOS sport and recreational	Pearson correlation	.075^2^	.123^2^	.375^2^
	*p*-value	.571	.350	.003
KOOS QoL	Pearson correlation	−.067^1^	−.115^1^	.184^1^
	*p*-value	.606	.379	.157
EQ-5D-3 L index	Pearson correlation	.172^2^	.134^2^	.109^2^
	*p*-value	.190	.306	.408
30-second chair stand test	Pearson correlation	.127^1^	.338^1^	.423^2^
	*p*-value	.331	.008	<.001

Superscript numbers indicate missing values

*EQ-5D-3 L* EuroQol quality of life assessment, *KOOS* knee injury osteoarthritis outcome score, *MVPA* moderate to vigorous physical activity, *PALS* physical activity level scale

### As-treated analysis

In line with the intention-to-treat analysis, the as-treated analyses showed no group differences for sensor-based physical activity. Contrary to the intention-to-treat analysis, the as-treated analysis showed no significant differences between treatment groups in self-reported physical activity.

### Drop out analysis

There were no differences between participants in the present activPAL cohort (*n* = 62) and participants who participated in in the original cohort but not included in the present cohort (*n* = 88) based on the following baseline variables: age, sex, radiographic OA, symptomatic OA, KOOS and EQ-5D-3 L index (*p* >.05). However, more participants reported they engaged in moderate to high physical activity at baseline compared to the non-participants (48% vs.20%, *p* <.001).

## Discussion

This study found no significant differences in accelerometer-assessed physical activity between the surgery and non-surgery group at 10-year follow-up. However, self-reported physical activity levels were higher in the non-surgery group compared to the surgery group as analysed with the intention-to-treat approach. Though, using the as-treated approach, there were no group differences in any of the physical activity variables. Positive correlations were observed between the self-reported physical activity and the KOOS_PAIN_, KOOS_ADL_ and KOOS_SPORT and RECREATION_, while accelerometer-measured time spent engaged in MVPA did not correlate with any of the PROMs. Individuals who walked more steps per day and reported higher levels of physical activity demonstrated better functional performance. Encouraging patients to increase their daily step count can improve functional performance, highlighting the importance of promoting physical activity in treatment plans for those with degenerative meniscus lesions.

Study strengths include long-term follow-up of a randomized controlled trial and using both self-reported and objective physical activity measures. About half of the individuals had symptomatic OA at the 10-year follow-up, potentially affecting their activity levels, so ANCOVA analyses were adjusted for symptomatic OA. Accelerometry provides consistent results and detailed information on activity intensity and duration [13], reducing biases like recall and social desirability. Sensor-based measurements offer reliable insights into movement patterns with high sensitivity and specificity [14, 15]. Our procedure calculated total knee load by adding data from all standing positions and distinguished sitting and lying from standing with high accuracy. By placing the sensor on the thigh, the static inclination of the thigh could be detected, which was used to distinguish sitting and lying from standing [30]. We assessed physical activity 24 h a day for a comprehensive view of movement patterns.

The sample size was relatively small, which may Limit the generalisability of our findings. However, dropout analyses revealed no differences in baseline variables between participants and non-participants. The physical activity data indicated that this was an active group. It should be noted that participants were more physically active than non-participants, suggesting that physically active individuals may be more inclined to wear an accelerometer to register their physical activity compared to those who are less physically active. The higher prevalence of radiographic OA in the non-surgery group at baseline could have influenced the results, although no significant group differences in radiographic OA, symptomatic OA or other patient characteristics were observed at the 10-year follow-up. While accelerometers provide objective data, they may not capture all types of physical activity, such as swimming or cycling. Measurement of physical activity for one week may not be representative of the actual physical activity behaviour. Moreover, all the patients did not have complete accelerometry data comprising seven full days of recording. To reduce the risk of including non-representative accelerometer recordings, we included only recordings with at least three 24-hour episodes. When comparing results between studies, it is important to acknowledge that accelerometer data processing settings may affect the results [31, 32]. To minimize participant burden and maintain feasibility within the clinical assessment protocol, functional performance was assessed using the 30-second chair stand test, although a broader test battery or composite index may have offered a more comprehensive evaluation, particularly for walking-related function.

In the intention-to-treat analysis, self-reported physical activity was higher in the non-surgery group at the 10-year follow-up, with two-thirds reporting moderate to high activity compared to one-third in the surgery group. This suggests non-surgery patients perceived themselves as more active. The as-treated analysis showed no significant differences between groups, indicating the self-reported activity differences were not due to treatment effects. Knee function did not differ between groups at the 10-year follow-up [7]. The intention-to-treat approach preserved randomisation benefits but reduced sensitivity, while the as-treated approach accounted for crossovers.

Positive correlations between self-reported physical activity and the KOOS subscales for pain, daily activities, and sport highlight the benefits of physical activity on knee health and quality of life. However, MVPA did not correlate with any of the PROMs. Self-reports may influence each other; those reporting high activity likely experience less pain and fewer restrictions, while those reporting low activity may have more pain and limitations. The finding that accelerometer-assessed physical activity did not correlate with the PROMs agrees with previous data [19] Accelerometer data provides detailed activity intensity and duration [13], while PROMs offer a broader view of knee function and habits. MVPA did not correlate with the 30-second chair stand test, but the number of steps per day did, indicating different measurements capture various aspects of physical activity and function. Steps per day may better reflect overall mobility and daily functional capacity.

Engaging in 150 min of physical activity per week is crucial for preventing illnesses. A systematic review showed that moving from inactivity to this level reduces cardiovascular disease by 17% and type 2 diabetes by 26% [10]. Accelerometer data revealed no significant differences in MVPA between treatment groups, with both achieving the recommended threshold of ≥ 150 min per week. These findings indicate that arthroscopic knee surgery does not contribute to improved long-term physical activity levels which aligns with previous findings showing similar long-term outcomes for non-surgery and surgery groups in terms of knee pain, symptoms, radiographic findings, and clinical status [7], as well as OA progression [8]. 

This study has important clinical implications. The absence of significant differences in long-term accelerometer-assessed physical activity levels between the surgery and non-surgery groups, coupled with higher self-reported physical activity in the non-surgery group, suggests that knee arthroscopy combined with exercise therapy does not provide additional benefits over exercise therapy alone. The present results align with current treatment guidelines that recommend non-surgical interventions such as exercise therapy as primary treatments for managing degenerative meniscus tears [33]. The correlations between high self-reported physical activity and less pain and better function in daily activities as well as in sports and recreational pursuits emphasise the need for healthcare providers to encourage physical activity in this population to improve knee health and overall quality of life. There are indications that physical activity may slow down the progression of structural knee joint disease. In women, moderate physical activity has been shown to be associated with less cartilage thickness loss compared to low physical activity [34]. Additionally, increasing physical activity is an important self-management strategy to decrease the risk of future cardiovascular disease [35]. Physical activity interventions for patients with knee OA show short-term improvement, but their effectiveness tends to decline over time [36], suggesting that patients may need continued support to maintain healthy physical activity habits. Future research should explore strategies to increase patients’ commitment to engaging in physical activity and investigate the long-term impact of different treatment approaches on physical activity and overall health. Understanding the factors that contribute to sustained physical activity can inform targeted interventions to support healthy behaviours and improve outcomes for individuals with degenerative meniscus lesions. The present findings underscore the complexity of measuring physical activity and indicate that a combination of objective and self-reported measures is needed to provide a more comprehensive understanding of an individual’s physical activity and functional status.

## Conclusion

Sensor-based physical activity levels did not differ between treatment groups. However, higher self-reported physical activity was associated with less pain and better function in daily activities as well as in sports and recreational pursuits. Furthermore, participants who walked more steps per day and reported higher levels of physical activity demonstrated better functional performance. These findings highlight the importance of promoting physical activity in this population to enhance knee health and overall quality of life.

## Data Availability

Availability of data and materialsData are available upon reasonable request.

## Appendix 1. processing of the activPAL data

Processing of the activPAL data The activPAL event file was downloaded from the sensor and loaded into MATLAB R2019a (MathWorks Inc., Nattick (MA), USA). Only days with < 95% of the time spent in the most prominent activPAL code (sitting, standing or stepping) with ≥ 500 steps were considered valid [37]. For each participant, as many valid 24-hour recording periods as possible were extracted and analysed, but only participants with at least 72-hours of data were included in the analysis.

Initially, time spent lying down was classified using a published algorithm [37]. This algorithm identified the longest sitting bout between consecutive noons as lying down, and extended this classification if it was directly preceded or followed by another prolonged sitting bout. The remaining time was then categorized into one of the following mutually exclusive categories, in the specified order:

1. Moderate to vigorous physical activity (MVPA) ≥ 10 min: Instances with activPAL activity intensity ≥ 3 metabolic equivalents of tasks (METs) for ≥ 10 min, allowing for 20% of data < 3 METs [38]
2. Total MVPA: Instances with activPAL activity intensity ≥ 3 METs for ≥ 1 min, allowing for 20% of data < 3 METs. MVPA was analysed in two bout lengths to reflect previous (≥ 10 min) and current (no minimal length) physical activity guidelines [28].
3. Light-intensity physical activity (LIPA): Remaining stepping bouts
4. Standing: Remaining standing bouts
5. Sitting: Remaining sitting bouts

Time spent in each behaviour including knee-loading activities was expressed in both minutes and % per day. Knee-loading activities included standing, LIPA, and total MVPA. Total steps measured by activPAL during each 24-hour-episode were averaged for each individual

## Abbreviations

ADL: Activities of daily living
ANCOVA: Analysis of covariance
CI: Confidence interval
EQ-5D-3L: The EuroQol Quality of Life Questionnaire
IQR: Interquartile range
KL: Kellgren & Lawrence Score
KOOS: The Knee injury and Osteoarthritis Outcome Score
LIPA: Light-intensity physical activity
METs: Metabolic equivalents of tasks
MVPA: Moderate to vigorous physical activity
OA: Osteoarthritis
PALS: Physical Activity Level Scale
PROMs: Patient-reported outcome measures
SD: Standard deviation
QoL: Quality of life
